# Changes in circumpapillary retinal nerve fiber layer thickness after vitrectomy for rhegmatogenous retinal detachment

**DOI:** 10.1038/s41598-022-13070-y

**Published:** 2022-06-10

**Authors:** Young Hoon Hwang, Zee Yoon Byun, Daniel Duck-Jin Hwang

**Affiliations:** 1 Department of Ophthalmology, Chungnam National University Hospital, Daejeon, Republic of Korea; 2 Department of Ophthalmology, Hangil Eye Hospital, #35 Bupyeong-daero, Bupyeong-gu, Incheon, 21388 Republic of Korea; 3 Department of Ophthalmology, Catholic Kwandong University College of Medicine, Incheon, Republic of Korea

**Keywords:** Retinal diseases, Optic nerve diseases

## Abstract

The study aimed to evaluate the long-term changes in circumpapillary retinal nerve fiber layer (RNFL) thickness after vitrectomy for rhegmatogenous retinal detachment (RRD) repair. A total of 33 eyes of 33 patients were enrolled. By using optical coherence tomography, the circumpapillary RNFL thickness was measured before surgery and 1, 3, 6 months and 1, 2, 3 years after surgery and compared with the preoperative value. The effect of duration, location, and extent of RRD on RNFL thickness change was evaluated. There was a significant increase of circumpapillary RNFL thickness at the 1-month, 3-month [except in the nasal superior sector (*P* = 0.627)], and only in the nasal inferior sector at 6-month (*P* = 0.010) follow-up compared with the baseline value (all *Ps* < 0.05). No significant differences were observed 1, 2, and 3 years after the surgery (*P* > 0.05). The duration, location, and extent of detachment did not reveal significant correlations with RNFL parameters (*P* > 0.05). Circumpapillary RNFL thickness in eyes with RRD after vitrectomy demonstrated a transient increase during the early postoperative period. This increase was not associated with duration, location, and extent of RRD. At 3 years following surgery, no RNFL thinning or thickening was observed.

## Introduction

Rhegmatogenous retinal detachment (RRD) is a vision-threatening condition characterized by separation of the neurosensory retina from the retinal pigment epithelium (RPE) with the presence of a retinal tear. Detachment and reattachment of the retina may induce changes in various retinal tissues, including the RPE, photoreceptors, bipolar cells, horizontal cells, glial cells, and retinal ganglion cells (RGCs)^1,2^. Given that the retinal nerve fiber layer (RNFL) comprises axons of RGCs, it can be speculated that RRD may cause changes in the RNFL thickness^3–11^. Retinal detachment involving the macular area can cause visual impairment. Thus, surgical intervention is required to repair the detached retina. In addition to RRD per se, surgical interventions, such as vitrectomy with or without injection of gas or silicone oil into the vitreous cavity, have also been reported to cause structural changes in the retinal layers^12–14^.

It has been hypothesized that RGC and RNFL thickness, as well as the structural integrity of outer retinal layers, may be associated with postoperative visual outcomes in eyes with RRD^4,8^. Therefore, the assessment of changes in the RNFL thickness after surgery for RRD may provide useful clinical information. To date, only a few studies have investigated the RNFL thickness in eyes with RRD, and these studies are affected by heterogeneous study designs and inconsistent results; RNFL thinning was found in several studies^4,8^, whereas RNFL thickness increase^5–7,10,11^ or no significant change^3^ was found in other studies. This study was performed to evaluate the longitudinal long-term changes in circumpapillary RNFL thickness after vitrectomy for RRD repair.

## Results

During the enrollment period, 35 eyes with RRD met the inclusion criteria. Among them, two eyes with segmentation errors in circumpapillary RNFL thickness detection were excluded. Thus, a total of 66 eyes from 33 patients (19 females, 14 males) with unilateral RRD were finally included. During this period, all cases included in the study underwent pars plana vitrectomy (PPV) with intravitreal C3F8 gas injection. The surgical success rate was 100%, and there was no case of reoperation during the follow-up period. Mean age was 54.2 ± 9.9 years (range, 19–70). Mean axial lengths of the affected and unaffected fellow eyes at baseline visit were 24.66 ± 1.31 mm (range, 23.00–27.95) and 24.70 ± 1.41 mm (range, 22.81–28.24), respectively (*P* = 0.109). Median (interquartile range) duration of detachment was 7.0 (4.5–14.5) days. Median (interquartile range) visual acuity of the affected eye at the baseline visit was 0.155 (0.046–0.301). Among the 33 eyes with RRD, 8 (24.2%) showed macular-off RRD. The extent of RRD was greater than 6 h in 8 of the 33 (24.2%) eyes and the location of RRD was most frequently in the superior quadrant (16 eyes, 48.5%), followed by temporal (7 eyes, 21.2%), nasal (6 eyes, 18.2%), and inferior (4 eyes, 12.1%) quadrants (Table 1).

**Table 1:** Preoperative baseline clinical characteristics of eyes with retinal detachment (n = 33).

Clinical parameters	Observed values
Age (years, mean ± SD)	54.2 ± 9.9
Female:male	19:14
Macular-on:macular-off	25:8
Right:left eye	19:14
**Extent of retinal detachment**	
3 to 6 h	25 (75.8%)
7 to 9 h	8 (24.2%)
**Location of retinal detachment**	
Superior	16 (48.5%)
Nasal	6 (18.2%)
Inferior	4 (12.1%)
Temporal	7 (21.2%)
Phakic:pseudophakic	24:9
Axial length (mm, mean ± SD)	24.66 ± 1.31
Visual acuity (logMAR, median, interquartile range)	0.155 (0.046–0.301)
IOP (mmHg, median, interquartile range)	15.0 (12.0–17.0)

Values are presented as number, mean ± standard deviation, or median (interquartile range).

*SD* Standard deviation, *logMAR* Logarithm of the minimum angle of resolution, *IOP* Intraocular pressure.

### Changes in visual acuity and intraocular pressure

Median (interquartile range) best corrected visual acuity (BCVA) of eyes with RRD (0.046, 0–0.155) significantly improved one year after the surgery compared with the baseline values (0.155, 0.046–0.301, *P* = 0.002). It remained significantly different from the baseline BCVA 2 and 3 years after the surgery (*P* = 0.010 and 0.006, respectively, Table 2). When the intraocular pressure (IOP) in eyes with RRD before the surgery was compared to that after, no significant difference was found at any visit (all *Ps* > 0.05). No eye showed an IOP rise greater than 21 mmHg during the follow-up. Eyes with RRD and unaffected fellow eyes did not show significant differences in IOP at any visit (all *Ps* > 0.05).

**Table 2:** Visual acuity and intraocular pressure change after surgery.

	Visual acuity (logMAR)	*P* value*	Intraocular pressure (mmHg)	*P* value*
Baseline	0.155 (0.046–0.301)		15.0 (12.0–17.0)	
1 month	0.222 (0.097–0.374)	0.042	15.0 (13.0–18.5)	0.218
3 months	0.097 (0.046–0.155)	0.432	14.0 (13.0–16.0)	0.736
6 months	0.046 (0–0.155)	0.049	14.0 (12.0–16.25)	0.550
1 year	0.046 (0–0.155)	0.002	15.0 (13.0–17.0)	0.878
2 years	0.072 (0–0.112)	0.010	15.0 (13.0–17.0)	0.943
3 years	0.046 (0–0.126)	0.006	15.0 (13.0–17.5)	0.304

Data are presented as median (interquartile range).

*logMAR* Logarithm of the minimum angle of resolution.

*Wilcoxon signed-rank test (compared with baseline value).

### Longitudinal change in circumpapillary retinal nerve fiber layer thickness after surgery

**Figure 1:**
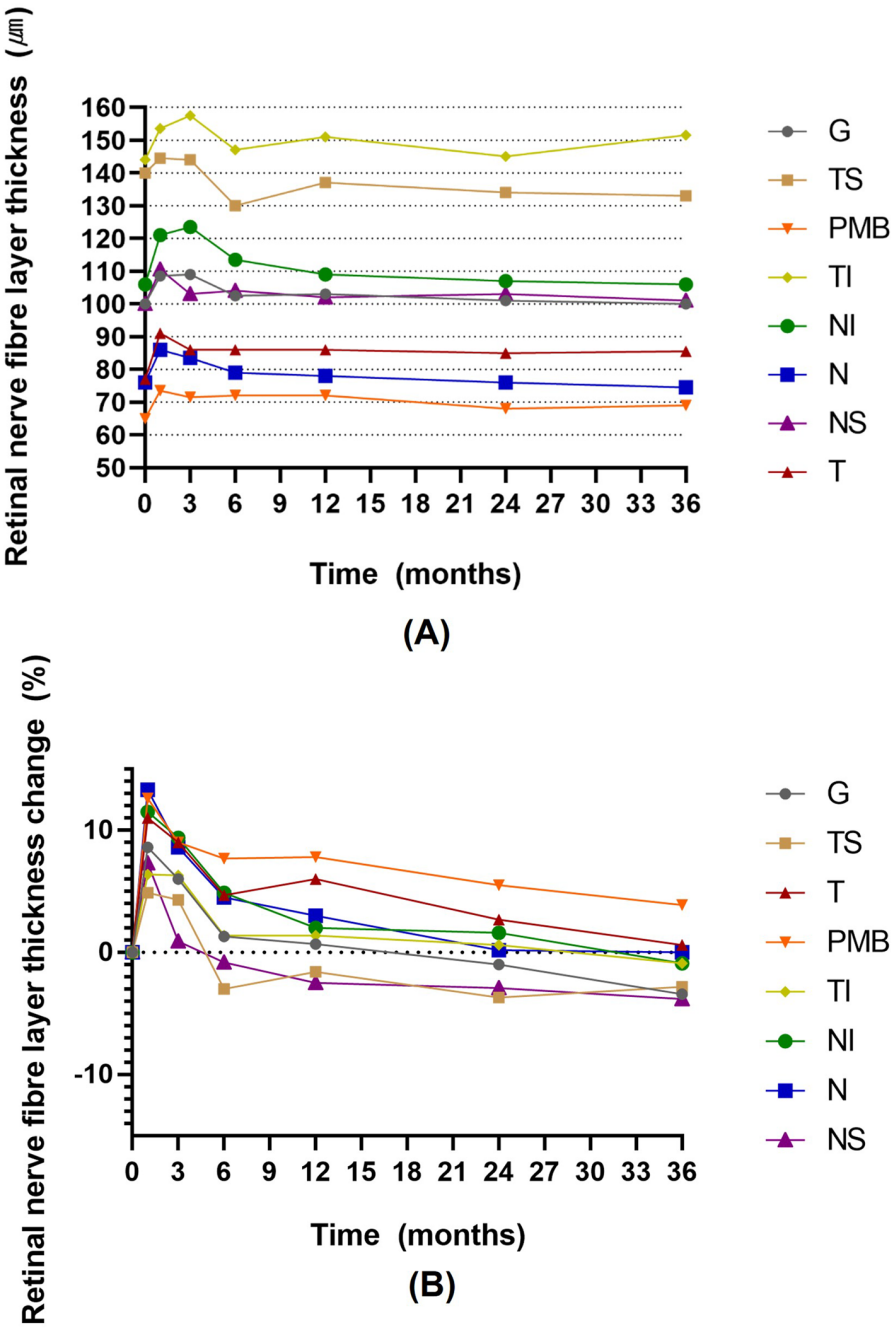
(**A**) Changes in retinal nerve fiber layer (RNFL) thickness of global area (G), temporal superior (TS), temporal inferior (TI), nasal superior (NS), nasal inferior (NI), temporal (T), nasal (N) sectors, and the papillomacular bundle (PMB) in eyes with rhegmatogenous retinal detachment after vitrectomy. (**B**) Relative changes presented as (RNFL thickness at follow-up visit—RNFL thickness at baseline visit/RNFL thickness at baseline visit) × 100 in G, TS, TI, NS, N, T, N sectors, and PMB in eyes with rhegmatogenous retinal detachment after vitrectomy.

For the eyes with detachment, there was a significant increase in circumpapillary RNFL thickness in all sectors at the 1-month follow-up compared with the baseline values (all *Ps* < 0.05). This change remained significant at the 3-month follow-up in all sectors (all *Ps* < 0.05) except in the NS sector (*P* = 0.627). At 6 months after the surgery, only the NI sector showed a significant difference (*P* = 0.010) and no significant difference was found in other sectors (all *Ps* > 0.05). No significant difference was found in all sectors 1, 2, and 3 years after the surgery compared with the baseline values (all *Ps* > 0.05, Table 3). Mean relative RNFL thickness change ranged from 4.9% (TS sector) to 13.3% (N sector) at the 1-month follow-up, from 0.9% (NS sector) to 9.4% (NI sector) at the 3-month follow-up, and from 0.6% (T sector) to − 3.8% (NS sector) at the 3-year follow-up (Fig. 1).

**Table 3:** Circumpapillary retinal nerve fiber layer thickness changes after surgery.

	Global	*P* value^†^	TS	*P* value^†^	T	*P* value^†^	PMB	*P* value^†^	TI	*P* value^†^	NI	*P* value^†^	N	*P* value^†^	NS	*P* value^†^
Baseline	100.0 (95.5–109.0)		140.0 (122.5–151.5)		77.0 (77.0–87.5)		65.0 (59.0–75.0)		144.0 (135.0–156.5)		106.0 (91.0–131.5)		76.0 (66.5–87.0)		100.0 (89.0–113.5)	
1 month	**108.5 (104.0**–**118.5)**	0.010*	**144.5 (118.25**–**158.75)**	0.037*	**91.0 (79.5**–**98.0)**	0.002*	**73.5 (67.5**–**84.25)**	0.002*	**153.5 (142.25**–**168.0)**	0.001*	**121.0 (100.0**–**141.25)**	< 0.001*	**86.0 (74.5**–**98.5)**	< 0.001*	**110.5 (97.75**–**122.5)**	0.004*
3 months	**109.0 (100.0**–**117.75)**	0.010*	**144.0 (133.00**–**152.75)**	0.017*	**86.0 (74.5**–**93.0)**	0.016*	**71.5 (63.75**–**77.5)**	0.040*	**157.5 (140.75**–**172.75)**	0.020*	**123.5 (108.0**–**142.25)**	< 0.001*	**83.5 (73.5**–**98.0)**	0.040*	108.0 (96.5–118.75)	0.627
6 months	102.5 (93.75–113.25)	0.415	130.0 (113.75–144.5)	0.461	86.0 (77.0–62.25)	0.518	72.0 (61.5–82.0)	0.130	147.0 (133.75–159.5)	0.306	**113.5 (88.50**–**138.25)**	0.010*	79.0 (67.25–90.5)	0.075	104.0 (86.5–112.75)	0.929
1 year	103.0 (97.0–112.5)	0.650	137.0 (125.0–142.5)	0.269	86.0 (75.0–97.0)	0.179	72.0 (62.25–82.25)	0.165	151.0 (136.0–158.5)	0.147	109.0 (91.5–130.5)	0.209	78.0 (67.0–87.5)	0.393	102.0 (85.0–111.5)	0.569
2 years	101.0 (92.0–109.0)	0.976	134.0 (117.0–145.0)	0.196	85.0 (71.0–98.0)	0.903	68.0 (61.25–80.75)	0.414	145.0134.0–160.0)	0.394	107.0 (82.0–129.0)	0.867	76.0 (68.0–87.0)	0.884	103.0 (87.5–108.0)	0.140
3 years	100.0 (93.0–109.0)	0.089	133.0 (117.25–144.75)	0.121	85.5 (71.25–96.5)	0.926	69.0 (56.5–83.5)	0.844	151.5 (132.0–154.75)	0.513	106.0 (89.25–133.0)	0.350	74.5 (62.75–87.25)	0.490	101.0 (91.25–107.0)	0.103

Significant values are in bold.

Data are presented as median (interquartile range).

*G* Global area, *TS* Temporal superior, *TI* Temporal inferior, *NS* Nasal superior, *NI* Nasal inferior, *T* Temporal, *N* Nasal, *PMB* Papillomacular bundle.

*Denotes a significant difference (*P* < 0.05).

^†^Wilcoxon signed-rank test (compared with baseline value).

The circumpapillary RNFL thicknesses of the fellow eye at 6 months, 1 year, 2 years, and 3 years after the surgery did not show significant change compared with baseline values in all sectors (all *Ps* > 0.05). When compared with the eyes with RRD, fellow eyes had thinner RNFL in NI (from baseline to 2-year follow-up) and N sectors (from baseline to 3-year follow-up, all *Ps* < 0.05, Table 4).

**Table 4:** Circumpapillary retinal nerve fiber layer thickness in fellows eyes of the eyes with retinal detachment.

	Global	*P* value^†^	TS	*P* value^†^	T	*P* value^†^	PMB	*P* value^†^	TI	*P* value^†^	NI	*P* value^†^	N	*P* value^†^	NS	*P* value^†^
Baseline	**98.0 (89.25**–**106.75)**	0.036*	133.5 (122.25–141.0)	0.651	77.5 (71.0–81.0)	0.504	**59.0 (56.0**–**66.0)**	0.014*	150.5 (136.0–156.75)	0.229	**101.0 (83.25**–**121.25)**	0.011*	**67.0 (60.0**–**75.5)**	0.001*	108.0 (88.5–119.75)	0.930
6 months	95.0 (89.5–108.5)	0.133	133.0 (114.5–147.0)	0.399	77.0 (71.0–85.5)	0.065	**61.0 (54.5**–**66.5)**	0.008*	148.0 (134.0–157.5)	0.600	**102.0 (81.5**–**113.0)**	0.021*	**66.0 (59.25**–**78.75)**	0.010*	106.0 (88.5–115.0)	0.742
1 year	99.5 (90.0–107.75)	0.167	134.0 (126.5–140.5)	0.799	78.0 (72.25–87.5)	0.210	60.0 (56.0–68.0)	0.066	150.0 (137.25–158.75)	0.683	**101.0 (87.25**–**112.75)**	0.007*	**67.0 (60.0**–**78.5)**	0.005*	110.5 (91.0–121.75)	0.178
2 years	99.0 (86.0–108.0)	0.229	130.0 (105.0–141.0)	0.649	85.0 (74.0–91.0)	0.935	66.0 (59.5–75.5)	0.222	145.0 (136.0–157.0)	0.820	**95.0 (83.0**–**122.0)**	0.033*	**70.0 (63.0**–**78.0)**	0.006*	100.0 (84.0–121.0)	0.770
3 years	100.0 (99.0–110.75)	0.896	132.5 (115.25–149.25)	0.970	83.0 (76.5–94.75)	0.354	67.0 (60.5–80.25)	0.670	149.0 (138.0–160.0)	0.184	101.0 (86.25–120.75)	0.112	**65.5 (57.5**–**77.5)**	0.002*	103.0 (86.5–121.5)	0.370

Significant values are in bold.

Data are presented as median (interquartile range) of fellow eyes.

*Denotes a significant difference (*P* < 0.05).

^†^Wilcoxon signed-rank test (comparison between eyes with retinal detachment presented in Table 3 and fellow eyes).

*G* Global area, *TS* Temporal superior, *TI* Temporal inferior, *NS* Nasal superior, *NI* Nasal inferior, *T* Temporal, *N* Nasal, *PMB* Papillomacular bundle.

### Affecting factors for circumpapillary retinal nerve fiber layer thickness change after surgery

Comparison of RNFL thickness and relative RNFL thickness change at each visit between eyes with superior RRD and inferior RRD and between eyes with macular-on RRD and macular-off RRD showed no significant differences at all sectors and visits (all *Ps* > 0.05). The duration, extent of detachment, and final BCVA did not show significant correlations with SD-OCT parameters (all *Ps* > 0.05).

## Discussion

In the present study, circumpapillary RNFL thickness in eyes with RRD after vitrectomy showed a transient increase during the early postoperative period (within 6 months) regardless of duration, location, and extent of RRD. This increase disappeared after this point and no RNFL thinning was found compared to the preoperative values until 3 years after the surgery. To the best of our knowledge, this is the only prospective long-term study investigating and comparing circumpapillary RNFL thickness changes after vitrectomy to the preoperative values.

To identify the effect of RRD on retinal layers, observation of retinal thickness changes after the development of RRD without surgical intervention may be needed. However, given that untreated RRD can induce severe visual loss, this may be limited. In human eyes, several studies have reported RNFL thickness changes after the surgical repair of detachment by using non-invasive imaging devices, such as scanning laser polarimeter^3^ or OCT^4–11^. A study using time-domain OCT reported that RNFL thinning was not found within 6 months^4^. However, RNFL was thinner in the area of detached retina than fellow eyes 12 and 24 months postoperatively^4^. The authors suggested that this may have been caused by atrophy of RGCs and a subsequent decrease in RNFL thickness^4^. In contrast, other studies found no significant change^3^ or increase in RNFL thickness after repair of the detachment^5–7,10,11^. Our study also revealed a transient increase in RNFL thickness during the early postoperative period (within 6 months) that may be explained by RNFL edema, retinal ischemia, or Müller cell proliferation associated with retinal detachment^3–7,10,11^.

Transient circumpapillary RNFL thickness increase after repair of RRD may be explained by either the effect of RRD and/or the result of surgical procedures to repair it. We hypothesized that if RRD itself causes RNFL thickness change, the duration, location, or extent of RRD may be associated with the location or amount of RNFL thickness change. Regarding the topographic correlation between RRD location and RNFL thickness change, a study using time-domain OCT analyzed the clock-hour sector RNFL thickness of detached area and revealed that RNFL thickness at the detached area was lower than corresponding area of the fellow eye^4^. However, given that the pathway of RNFL is not linear, this method may not reflect the real topographic correlation^15^. The RNFL in superior or inferior hemisphere does not cross the horizontal raphe. Therefore, we compared the RNFL thickness change pattern between eyes with RRD in the superior and inferior hemispheres and found no significant association of the location of RRD with RNFL thickness change patterns. A study reported that RNFL thickness increase after retinal detachment was associated with increase in the duration of detachment; this may be caused by the proliferation of Müller cells, which may be more prominent in longstanding detachment^3^. If this were true, eyes with a longer duration of detachment may have a greater preoperative RNFL thickness. However, we could not find any significant correlation between the duration of detachment and OCT parameters. Furthermore, the extent of RRD was not correlated with the amount of RNFL thickness change. These findings imply that the transient RNFL thickness increase may not be directly related to the RRD itself.

It has been reported that transient RNFL thickness increase was also found after vitrectomy for macular hole or epiretinal membrane^12–14^. This may be caused by trauma to the inner retina, fluctuations of IOP, toxicity to the retina from light, dye, fluids, or gases, and inflammatory responses during the surgery^12–14^. During the vitrectomy for RRD, similar procedures and devices are applied to the eye. Therefore, we think that comparable mechanisms to vitrectomy for macular hole or epiretinal membranes may play a role in eyes with RRD during the vitrectomy.

When RNFL thickness of eyes with RRD was compared with the RNFL thickness of unaffected fellow eyes, no significant difference was found 1 year after the surgery, with the exception of RNFL in NI and N sectors; RNFL thicknesses in these sectors of affected eyes were greater than in the fellow eyes. This may be explained by a greater RNFL thickness of affected eyes in these areas before the development of RRD or a greater effect of vitrectomy on these areas compared to other areas. A previous study also reported that RNFL thickness increase after vitrectomy was most prominent in the nasal area^13^. Further studies with similar topographic characteristics of the RNFL between affected and unaffected eyes at preoperative baseline visit are required to elucidate on this issue.

Most of the previous studies with RRD evaluated RNFL thickness compared to the unaffected fellow eyes or eyes of healthy control participants^3–7,11^. Although the fellow eye may be a useful control, it has been reported that inter-eye differences in RNFL thickness exist within the same individual^16,17^. Therefore, preoperative baseline values of the same eye may be a better control than the fellow eye or healthy controls. A possible limitation of comparison with the preoperative baseline value is the presence of OCT segmentation errors or artifacts caused by RRD. To minimize this confounding effect, we only included eyes without RRD within the scan circle area centered on the optic nerve head.

It has been suggested that a thin RNFL may reflect worse visual function after RRD repair^4,8^. In the present study, final BCVA was not significantly associated with any RNFL thickness parameters. Previous studies also reported that thickness of the inner retinal layers did not show significant correlation with vision^7,10^. We investigated only RNFL thickness values measured where a 3.46-mm scan circle passes; RNFL thickness change distal to the 3.46-mm scan circle was not evaluated. It has been reported that when assessing RNFL thickness change in glaucoma, progression was most frequently found in the inferotemporal area outside of the 3.46-mm scan circle^18^. Therefore, we may speculate that RNFL thickness analysis in eyes with RRD including RNFL thickness values distal to the 3.46-mm scan circle may provide more information. In the present study, only thickness values were investigated. Assessment of structural integrity (i.e., presence of irregularity, discontinuity, or cystic change) other than thickness change should be considered. Visual function can be evaluated not only by BCVA but also by visual field test or electroretinogram. Further studies with these modalities may be needed. To conduct a prospective long-term follow-up study, it was difficult to enroll participants with a large number. A relatively smaller number of participants in the current investigation compared to the previous studies with 33 to 67 eyes^4–7,9,10^ may have caused a lower power to detect a clinically relevant change in the RNFL.

In conclusion, circumpapillary RNFL thickness in eyes with RRD after vitrectomy showed transient increase during the early postoperative period. This increase was not associated with duration, location, or the extent of RRD. Until 3 years after surgery, no RNFL thinning was found and the RNFL thickness values were not correlated with visual acuity after the surgery.

## Materials and methods

### Participants

This prospective cohort study protocol was approved by the Institutional Review Board of Hangil Eye Hospital. The study was carried out in accordance with the tenets of the Declaration of Helsinki. Informed consent was obtained from the participants. Among the patients with RRD who underwent PPV to repair detachment at Hangil Eye Hospital between February 2017 and June 2018, those aged ≥ 18 years and with RRD in the affected eye with a healthy fellow eye, no history of previous retinal surgery in either eye, absence of other retinal diseases (i.e., epiretinal membrane, diabetic retinopathy, retinal vein occlusion, and macular degeneration), optic neuropathy (i.e., glaucoma), or any condition with media opacity that can cause insufficient spectral domain optical coherence tomography (SD-OCT) image quality in either eye, were consecutively enrolled.

**Figure 2:**
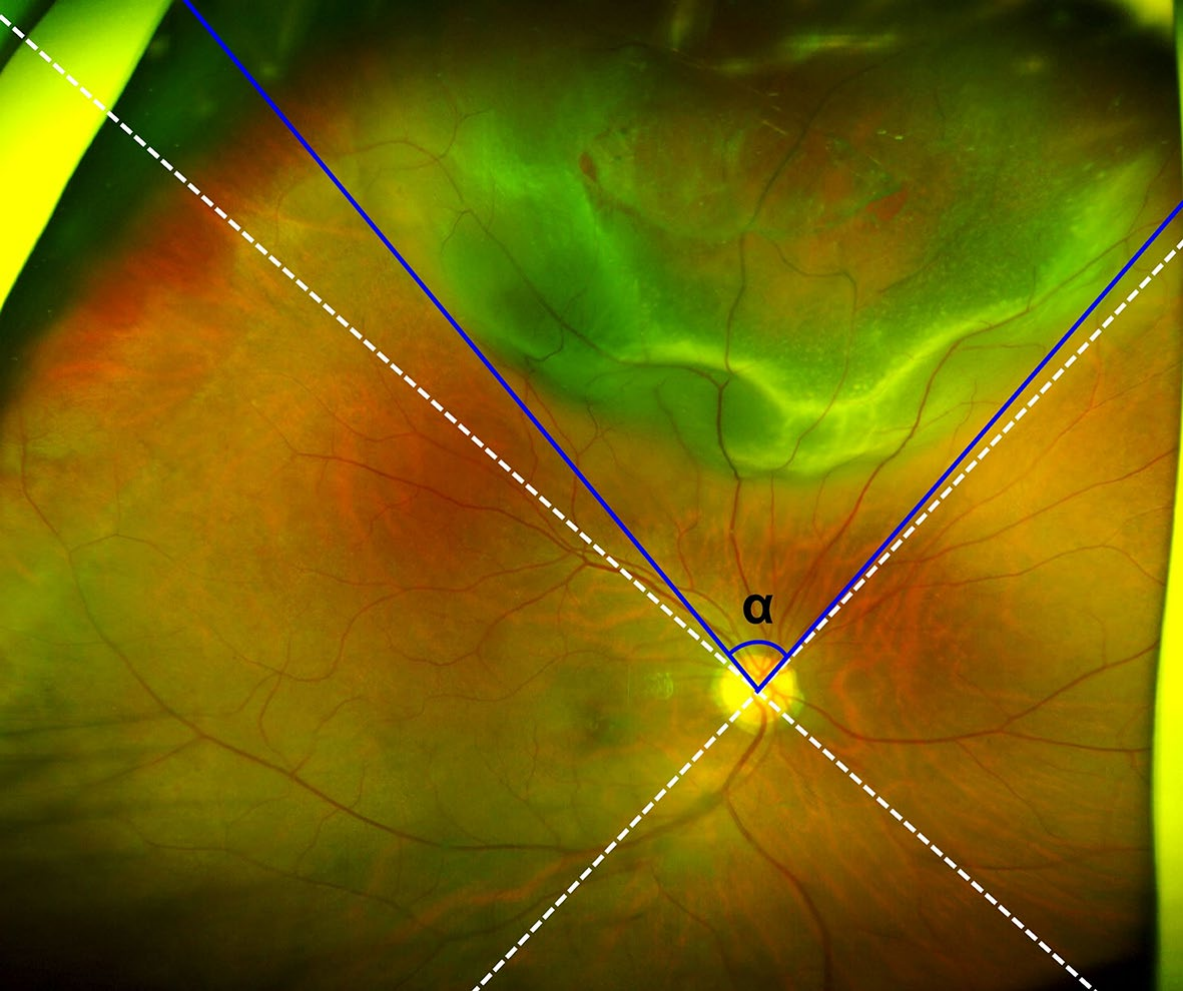
Definition of the location and extent of retinal detachment. The location was subdivided into superior, nasal, inferior, or temporal quadrants based on the imaginary lines centered on the optic nerve head (dotted lines). The extent was defined as the angular distance between lines connecting the optic nerve head center and the margins of detached retina (blue solid lines, angle α).

For the eyes with RRD, the location of the detachment was subdivided into superior, nasal, inferior, and temporal quadrants in accordance with the SD-OCT circumpapillary RNFL quadrant classification (dotted lines in Fig. 2). When it was present across multiple quadrants, the quadrant with the greatest area of detachment was selected. The extent of RRD was defined as the angular distance between the lines connecting the margins of the RRD to optic nerve head center (angle α in Fig. 2). The extent was presented as clock-hour area.

### Surgical methods

A single surgeon (DDH) performed all the surgeries. A 3-port 25-gauge scleral tunnel transconjunctival sutureless PPV was performed using the Constellation Vision Surgical System (Alcon Surgical, Fort Worth, TX, USA). It was performed under 2% lidocaine sub-Tenon anesthesia with monitored anesthesia care. Phacoemulsification with posterior chamber intraocular lens implantation was also performed in patients with cataract. After the core vitrectomy, the peripheral vitreous was thoroughly shaved using scleral indentation. The subretinal fluid was drained through the preexisting peripheral retinal breaks after filling perfluorocarbon liquid (PFCL, Decaline® 7 mL, FCI SAS, Paris, France) up to the posterior edge of the break. Endophotocoagulation around the breaks on the peripheral retina was then applied. Following PFCL removal and fluid-air exchange, the surgery was completed after confirmation of the reattachment of retina and absence of any epiretinal membrane and macular hole using the intraoperative OCT (RESCAN 700, Zeiss, Germany) in all patients. After that, in all cases, gas endo-tamponade (C3F8, 10% perfluoropropane) was performed and those patients were then recommended to maintain a prone position for one week after surgery.

### Ophthalmic examinations

Slit-lamp examination, fundoscopy, BCVA assessments presented as a logarithm of the minimum angle of resolution (logMAR), IOP measurements using Goldmann applanation tonometry, and circumpapillary RNFL thickness measurements were performed at the baseline visit before surgery and 1 month, 3 months, 6 months, 1 year, 2 years, and 3 years after surgery. The RNFL thickness of the fellow eye was measured at the baseline visit before surgery and 6 months, 1 year, 2 year, and 3 years after surgery. Axial length was measured by IOLMaster 700 (Carl Zeiss Meditec AG, Jena, Germany) at the baseline visit. Circumpapillary RNFL thickness was measured using SD-OCT (Spectralis OCT; Heidelberg engineering, Heidelberg, Germany). After proper alignment, the optic disc was centered to the SD-OCT scan and images were acquired. By using an equipped software (Spectralis Nsite Axonal Analytics Software; Heidelberg Engineering, Heidelberg, Germany), circumpapillary RNFL thickness was automatically segmented and presented in six sectors: temporal (T, 315°–45°), temporal superior (TS, 45°–90°), nasal superior (NS, 90°–135°), nasal (N, 135°–225°), nasal inferior (NI, 225°–270°), and temporal inferior (TI, 270°–315°), together with the papillomacular bundle (PMB, 338°–8°) thickness. The global (G) RNFL thickness was obtained by averaging the total 360° RNFL thicknesses. Given that detached retina within the SD-OCT scan area may cause a segmentation error, only eyes with retinal detachment not involving the 3.46-mm scan circle centered on the optic nerve head and eyes without segmentation errors or artifacts were included.

### Statistical analyses

Statistical analyses were performed using a commercially available software (SPSS Statistics version 23; IBM Corp., Armonk, NY, USA). Distribution of variables was evaluated by Kolmogorov–Smirnov test. Variables with a normal distribution were presented as mean and standard deviation, while variables without a normal distribution were presented as median and interquartile range, and categorical variables were described as frequencies. Visual acuity, IOP, and SD-OCT thickness values did not show a normal distribution. Thus, comparison between the baseline visit and each follow-up visit and the comparison between the affected eye and fellow eye at each visit were performed by the Wilcoxon signed-rank test. Comparison of axial lengths in the affected eye and fellow eye was performed by the paired *t*-test.

To investigate the effect of RRD location on RNFL thickness change, the eyes were subdivided into eyes with superior, nasal, inferior, and temporal RRD based on the RRD location. To identify the effect of macular status on RNFL thickness change, the eyes were subdivided into macular-on and macular-off groups. RNFL thickness and relative RNFL thickness change (%), presented as (RNFL thickness at follow-up visit—RNFL thickness at baseline visit/RNFL thickness at baseline visit) × 100, at each visit were compared between the groups using the Mann–Whitney U test. Correlation between the duration and extent of detachment, final BCVA, and SD-OCT parameters were assessed by Spearman’s correlation coefficient. A *P* value < 0.05 was considered statistically significant.

## Data Availability

The data are not available for public access because of patient privacy concerns, but are available from the corresponding author on reasonable request.
